# ﻿A new species of *Androctonus* from the Najd Plateau of Saudi Arabia (Scorpiones, Buthidae)

**DOI:** 10.3897/zookeys.1262.163047

**Published:** 2025-12-03

**Authors:** Ersen Aydın Yağmur, Abdulaziz R. Alqahtani, Ahmed Badry

**Affiliations:** 1 Manisa Celal Bayar University, Alaşehir Vocational School, Alaşehir, Manisa, 45600, Turkiye Manisa Celal Bayar University Alaşehir Turkiye; 2 Department of Biology, College of Science, University of Bisha, P.O. Box 551, Bisha 61922, Saudi Arabia University of Bisha Bisha Saudi Arabia; 3 Department of Zoology, Faculty of Science, Al-Azhar University, Nasr City, P.O. Box: 11751, Cairo, Egypt Al-Azhar University Cairo Egypt

**Keywords:** COI, description, molecular phylogeny, morphology, phylogenetic analysis

## Abstract

A new scorpion species, *Androctonus najdensis***sp. nov.**, is described and illustrated from Saudi Arabia. It is compared with existing species from the Middle East and Iran, notably *A. crassicauda* (Olivier, 1807) and the recently described *A. tihamicus* Alqahtani, Yağmur & Badry, 2023. Molecular analysis using the COI mitochondrial gene revealed a genetic divergence of 7.0 to 11% between *A. najdensis***sp. nov.** and *A. crassicauda* sensu lato samples from Saudi Arabia, Iraq, and Iran. The combination of molecular and morphological data supports the recognition of the Najd populations as a distinct species. Additionally, an identification key for *Androctonus* species in Iran and the Middle East is provided.

## ﻿Introduction

The genus *Androctonus* was established by Ehrenberg in Hemprich and Ehrenberg (1828) and is distributed across North Africa, the Middle East, and western Asia, with 54 valid species currently recognized (Fet and Lowe 2000; Abu Afifeh et al. 2025; Kovařík et al. 2025; Lowe 2025; Rein 2025; Yağmur et al. 2025c). This genus presents taxonomic challenges due to considerable intraspecific morphological and molecular variation (Ben Ali et al. 2000; Lourenço 2005, 2008; Lourenço and Qi 2006, 2007; Othmen et al. 2009; Alqahtani et al. 2022a, b). *Scorpio crassicauda* Olivier, 1807 was originally described from Kashan, Iran; Simon (1872) later transferred it to the genus *Buthus*, and Kraepelin (1891) subsequently classified it under *Androctonus*. *Androctonus crassicauda* sensu lato is widely distributed and known for its medical importance (Chippaux and Goyffon 2008; Alqahtani and Badry 2021). Its range extends from Sinai, Egypt, across the Arabian Peninsula and the Middle East to Iran (Crucitti 1999; Amr et al. 2021). The presence of *A. crassicauda* s.l. in Saudi Arabia has been reported by several authors (e.g., Vachon 1979; Hendrixson 2006; Al-Asmari et al. 2009, 2013; Alqahtani et al. 2019).

Alqahtani et al. (2022a, b, c) reported intraspecific morphological and molecular variation among *A. crassicauda* s.l. populations collected from different ecogeographical regions of Saudi Arabia, revealing the presence of three cryptic taxa within these populations. More recently, Alqahtani et al. (2023) described *A. tihamicus* Alqahtani, Yağmur & Badry from the Tihamah Plain, which had previously been considered part of *A. australis*. In Turkey, *A. turkiyensis* Yağmur, 2021 was described for the Şanlıurfa population; the author also designated a neotype for *A. crassicauda* s.s. from Kashan, Iran (Yağmur, 2021). Later, *A. kunti* Yağmur, 2023 was described from Iğdır Province, Turkey. Additionally, *A. sumericus* Khazali & Yağmur, 2023 from Dhi Qar Province, Iraq and *A. ishtar* Yağmur, Kachel, Al-Khazali, Al-Jubouri & Ali, 2025 from Dohuk and Nineveh provinces, Iraq, were subsequently described (Khazali and Yağmur 2023; Yağmur et al. 2025b). Recently, *A. ammoneus* Yağmur, Al-Saraireh & Abu Afifeh, 2025 and *A. minaeus* Abu Afifeh, Al-Saraireh & El-Hennawy, 2025 were described from Jordan (Abu Afifeh et al. 2025; Yağmur et al. 2025a), while *A. ammophilus* Lowe, 2025 and *A. omanensis* Lowe, 2025 were described from Oman and adjacent parts of Saudi Arabia and the United Arab Emirates (Lowe, 2025). However, the taxonomic status of the remaining *Androctonus* populations across the Middle East remains unresolved.

In Iran, Barahoei et al. (2022) reviewed *A. baluchicus* (Pocock, 1900) and described *A. sistanus* Barahoei & Mirshamsi, 2022 from Sistan and Baluchistan Province. Later, Barahoei et al. (2025b) reviewed *Androctonus* populations across Iran, described *A. rostami* Barahoei, Mirshamsi, Amiri, Moeinadini & Rakhshani, 2025, and recorded *A. sumericus*. In a separate study, Barahoei et al. (2025a) examined populations from East Azerbaijan Province and recorded both *A. kunti* and *A. turkiyensis*. Very recently, Yağmur et al. (2025c) reviewed *Androctonus* populations in Iran and redescribed *A. crassicauda* (Olivier, 1807); they removed *Prionurus crassicauda orientalis* Birula, 1900 from synonymy and elevated it to species rank as *A. orientalis* (Birula, 1900), synonymized *A. rostami* with this species, and described *A. azerianus* Yağmur & Kovařík, 2025 (West Azerbaijan and Zanjan provinces), *A. barahoeii* Kovařík & Yağmur, 2025 (Bushehr, Chahar Mahal & Bakhtiyari, Fars, Ilam, Khuzestan, Kohgilouyeh & Boyer Ahmad, Lorestan, and Markazi provinces), *A. caspius* Kovařík, Yağmur, Fet & Lowe, 2025 (Alborz and Tehran provinces), and *A. transcaucasicus* Kovařík, Yağmur, Fet & Lowe, 2025 (Armenia, Azerbaijan, Iran: East Azerbaijan and West Azerbaijan provinces). Yağmur et al. (2025c) did not detect either *A. kunti* or *A. turkiyensis* but considered the presence of *A. kunti* in Iran plausible due to the absence of any significant geographical barriers.

In this study, we examined the *Androctonus* population of the Najd Plateau, previously reported as *A. crassicauda*, and describe a new species, *A. najdensis* sp. nov. We compared this new species with *A. ammophilus*, *A. omanensis*, and *A. tihamicus* from Arabian Peninsula, as well as with *A. ammoneus*, *A. azerianus*, *A. barahoeii*, *A. caspius*, *A. crassicauda* (Olivier, 1807), *A. ishtar*, *A. kunti*, *A. minaeus*, *A. orientalis*, *A. sumericus*, *A. transcaucasicus* and *A. turkiyensis* from Iraq, Iran, Jordan, and Turkey.

## ﻿Materials and methods

A total of 30 specimens of *Androctonus najdensis* sp. nov. were collected at night using ultraviolet light in Ha’il Province, Al Qassim, Riyadh, and the Eastern Province in 2021 (Fig. 17). The specimens were preserved in 96% ethanol. Photographs were taken following the methodology described by Yağmur (2021). Trichobothrial nomenclature follows Vachon (1974), and morphological terminology follows Francke (1977), Stahnke (1971), and Hjelle (1990).

The male holotype and a female paratype of *A. najdensis* sp. nov. were deposited in the Alaşehir Zoological Museum, Manisa Celal Bayar University, Alaşehir, Manisa, Turkey (**AZMM**), and the Al-Azhar University Zoological Collection (**AUZC**), Nasr City, Cairo, Egypt.

The whole genomic DNA was isolated from nine samples collected from Najd Plateau from Hail, Riyadh, and Eastern provinces as previously reported by Alqahtani et al. (2022a). The sequences were edited using BioEdit v. 7.2.5 (Hall 1999). Additional sequences of *Androctonus* species were obtained from GenBank, as well as the sequence of *Scorpio palmatus* (AY156585.1) downloaded as the outgroup (Table 1). The sequences were aligned using ClustalW in Mega 11 (Tamura et al. 2021), and genetic distances (p-distances) for the entire data set were calculated using Mega 11 (Tamura et al. 2021).

**Table 1.** List of *Androctonus* samples collected from Saudi Arabia used in this study and GenBank sequences, and museum number/accession numbers of COI marker used in the phylogenetic analysis.

Species	Location	Region	Country	Lat., Long.	Museum number /Accession number	Reference
*A. crassicauda*	Arar	Northern Borders Province	Saudi Arabia	30.88, 40.87	2	This study
*A. crassicauda*	Arar	Northern Borders Province		30.88, 40.87	3	This study
*A. crassicauda*	Arar	Northern Borders Province		30.88, 40.87	4	This study
*A. crassicauda*	Dumah Al Jandal	Al Jowf		29.84, 39.73	52	This study
*A. crassicauda*	Dumah Al Jandal	Al Jowf		29.84, 39.73	42	This study
*A. crassicauda*	Dumah Al Jandal	Al Jowf		29.84, 39.73	5	This study
*A. najdensis* sp. nov.	Hail	Hail Province		27.37, 41.73	11	This study
*A. najdensis* sp. nov.	Hail	Hail Province		27.37, 41.73	12	This study
*A. najdensis* sp. nov.	Hail	Hail Province		27.37, 41.73	13	This study
*A. najdensis* sp. nov.	Buraydah	Al Qassim		26.241, 43.94	1	This study
*A. najdensis* sp. nov	Buraydah	Al Qassim		26.241, 43.94	44A	This study
*A. najdensis* sp. nov	Khurais	Eastern Province		25.07, 48.02	16	This study
*A. najdensis* sp. nov.	Khurais	Eastern Province		25.07, 48.02	41	This study
*A. najdensis* sp. nov.	Nazeem, east of Riyadh	Riyadh		24.86, 47.06	9	This study
*A. najdensis* sp. nov.	Dhurma	Riyadh		24.54, 46.17	16	This study
*A. crassicauda*	Mahneshan	Mahneshan	Iran	36.22, 47.86	MH352603	GenBank
*A. crassicauda*	Sari_Aghol	Mahneshan		36.95, 46.93	MH352604	GenBank
*A. crassicauda*	Sahand-e Olya	Mahneshan		36.77, 47.52	MH352605	GenBank
*A. crassicauda*	Darram	Zanjan Province		37.02, 48.77	MH352606	GenBank
*A. crassicauda*	Chavarzagh	Zanjan Province		36.80, 49.66	MH352607	GenBank
*A. crassicauda*	Taroom	Sansooz		35.49, 48.20	MH352608	GenBank
*A. crassicauda*	Zanjan	Zanjan Province		36.69, 48.50	MH352609	GenBank
*A. crassicauda*	Doasb	Zanjan Province		36.16, 48.95	MH352610	GenBank
*A. crassicauda*	Daneshgah	Zanjan Province		36.67, 48.50	MH352611	GenBank
*A. crassicauda*	Sardasht	West Azerbaijan Province		36.14, 45.47	MK814934	Soltan et al. 2021
*A. crassicauda*	Sardasht	West Azerbaijan Province		35.68, 45.19	MK814933	Soltan et al. 2021
*A. crassicauda*	–	–	Iraq	36.86, 42.98	MT229840	GenBank

Phylogenetic analyses of the COI data set (*n* = 34) were performed following Alqahtani and Badry (2020). Maximum-parsimony and neighbour-joining analyses were conducted with PAUP* v. 4 (Swofford 2001) using heuristic clustering based on TBR branch swapping. To assess the confidence within the nodes, 1000 bootstrapping replicates and random additions of taxa were used (Felsenstein 2002). The best-fit nucleotide evolution models were determined using MrModeltest v. 2.3 (Nylander 2004) based on the Akaike Information Criterion in PAUP* v. 4 (Swofford 2001). Bayesian inference (BI) was implemented using MrBayes v. 3.1.2 (Ronquist et al. 2012) for a million generations, and output parameters were plotted with Tracer v. 1.7 (Rambaut et al. 2018) to infer the geographic structure.

## ﻿Systematics

### ﻿Family Buthidae C. L. Koch, 1837

**Genus *Androctonus* Ehrenberg, 1828**

#### ﻿ Androctonus najdensis sp. nov.

Taxon classificationAnimaliaScorpionesButhidae

B1E2B7F2-E186-58F3-9572-14A74946EADC

https://zoobank.org/31020907-3DB3-4451-A3DE-3EC0047D4247

Figs 1, 2, 3, 4, 5, 6, 7, 8, 9, 10, 11, 12, 13, 14, 15; Table 2

Buthus crassicauda : Kraepelin 1899: 16–17.
Androctonus crassicauda : Vachon 1966: 210; Vachon 1979: 31–34; Levy and Amitai 1980: 23–29; Al-Safadi 1992: 96; El-Hennawy 1992: 97, 101, 109–110; Fet and Lowe 2000: 72–73; Hendrixson 2006: 38–43; Al-Asmari et al. 2007: 836; Al-Asmari et al. 2009: 100; Al-Asmari et al. 2013: 5–7; Alqahtani et al. 2019: 21–22; Alqahtani et al. 2022a: 171–179; Alqahtani et al. 2022b: 1–18; Alqahtani et al. 2022c: 1–7.

##### Type material.

***Holotype***: • ♂ Saudi Arabia, Al Ahsa Province, Khurais, 25°04'12"N, 48°01'12"E, 449 m a.s.l., 10.III.2021, leg. A. Alqahtani (AZMM/Sco-2021:25). ***Paratypes***: • Saudi Arabia, Hail, Almada’n, 27°22'12"N, 41°43'48"E, 1038 m a.s.l., 1 ♀, 10.III.2021, leg. A. Alqahtani (AZMM/Sco-2021:25); • 5 ♂, 27°22'12"N, 41°43'48"E, 1038 m a.s.l., 10.III.2021, leg. A. Alqahtani (AUZC/Sco-2021: 1103–1107). • Al Qassim, Buraydah 26°14'27"N, 43°56'24"E, 665 m a.s.l., • 2♀, 20.III.2021, leg. A. Alqahtani (AUZC/Sco-2021: 1108–1109). • 5 ♂, Hail, 27°22'12"N, 41°43'48"E, 1038 m a.s.l., 20.III.2021, leg. A. Alqahtani (AUZC/Sco-2021: 1110–1114). • Riyadh; 2♀, Dhurma, 24°32'23"N, 46°10'11"E, 628 m a.s.l., 5.IV.2021, leg. A. Alqahtani (AZMM /Sco-2021: 1115–1116). • Dhurma, 24°32'23"N, 46°10'11"E, 628 m a.s.l., 4 ♂, 5.IV.2021, leg. A. Alqahtani (AUZC/Sco-2021: 1117–1120). • Saudi Arabia, Al Ahsa Province, Khurais, 25°04'12"N, 48°01'12"E, 449 m a.s.l., 8♀, 10.IV.2021, leg. A. Alqahtani (AUZC/Sco-2021: 1121–1128). • Saudi Arabia, Al Ahsa Province, Khurais, 25°04'12"N, 48°01'12"E, 449 m a.s.l., 2 ♂, 10.IV.2021, leg. A. Alqahtani (AUZC/Sco-2021: 1129–1130).

**Table 2.** Comparative measurements of types of *Androctonus najdensis* sp. nov. Abbreviations: length (L), width (W, in carapace it corresponds to posterior width), depth (D).

Dimensions (mm)	Ratio	*Androctonus najdensis* sp. nov. ♂, holotype	*Androctonus najdensis* sp. nov. ♀, paratype
Carapace	L / W	12.26 / 10.56	11.79 / 11.63
Mesosoma	L	25.37	22.14
Tergite VII	L / W	7.77 / 10.22	6.57 / 11.40
Metasoma + telson	L	55.73	56.33
Segment I	L / W / D	7.70 / 7.34 / 6.15	7.98 / 7.08 / 6.14
Segment II	L / W / D	8.47 / 8.34 / 7.25	9.50 / 7.62 / 6.76
Segment III	L / W / D	9.62 / 9.43 / 8.17	9.26 / 8.56 / 7.50
Segment IV	L / W / D	10.31 / 9.31 / 8.41	9.90 / 7.93 / 7.40
Segment V	L / W / D	10.96 / 8.08 / 7.20	10.95 / 7.11 / 6.04
Telson	L / W / D	8.67 / 3.88 / 3.16	8.74 / 4.24 / 3.16
Pedipalp	L	36.00	37.88
Femur	L / W	8.41 / 3.03	8.80 / 2.78
Patella	L / W	10.07 / 4.21	10.67 / 4.08
Chela	L	17.52	18.41
Manus	L / W / D	7.72/ 5.38 / 4.77	8.03 / 5.44 / 4.74
Movable finger	L	11.67	11.44
Fixed finger	L	9.01	8.83
**Total**	**L**	**93.36**	**90.26**

##### Diagnosis.

Medium-sized scorpions. Average body length 73 mm in males and 79 mm in females. General coloration ranges from dark brown to blackish brown. Carapace carinae strong and bear coarse granules in males, and moderate granules in females. Intercarinal areas densely covered with coarse to medium granules in males, and with medium to minute granules in females. Chela manus smooth and lustrous; internal surface densely granular in males and sparsely granular in females. Movable fingers of the pedipalps bear 16 rows of denticles, with external and internal accessory denticles and three distal granules. Fixed fingers bear 15 rows of granules, also with external and internal accessory granules and three distal granules. Trichobothrium *et* located between *dt* and *est*, and proximal to *dt*. Pectines bear 30–35 teeth in males and 23–28 in females. Tergites I–VI densely granular. All metasomal segments robust, longer than wide, and wider than deep. Segment V considerably more robust in males. Segments I–III have ten carinae, segment IV has eight, and segment V has five carinae. Dorsolateral carinae strong, with granules increasing in size posteriorly on segments I–IV; serrate on segment I, serrate to dentate or subdentate on segment II, and dentate on segments III–IV. On segment V, dorsolateral carinae strong, smooth anteriorly, and serrate posteriorly. Ventrolateral carinae strong on segments I–IV, with moderate and rounded granules on segments I and II, and coarse, rounded granules on segments III and IV. On segment V, ventrolateral carinae strong, bearing somewhat large granules that gradually and slightly increase in size posteriorly, without enlarged denticles.

##### Description.

**Figure 1.**
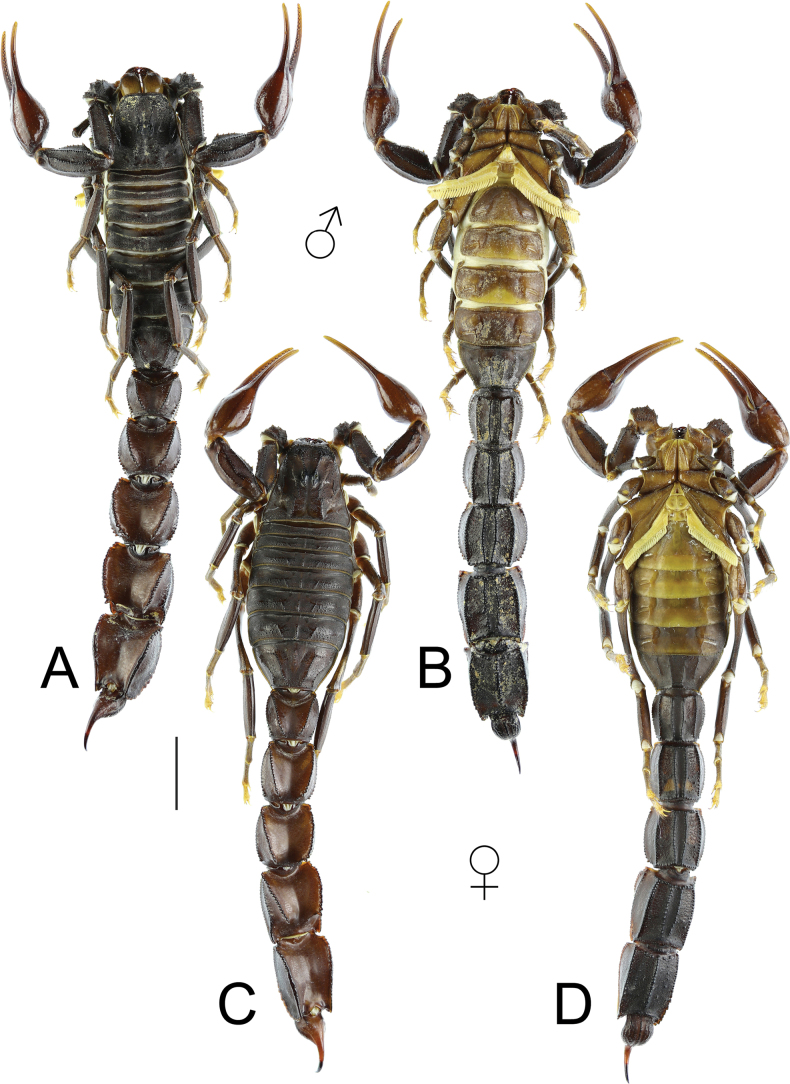
Habitus of *Androctonus najdensis* sp. nov., male holotype and female paratype. A, B. Male; C, D. Female; A, C. Dorsal view; B, D. Ventral view. Scale bar: 10 mm.

**Figure 2.**
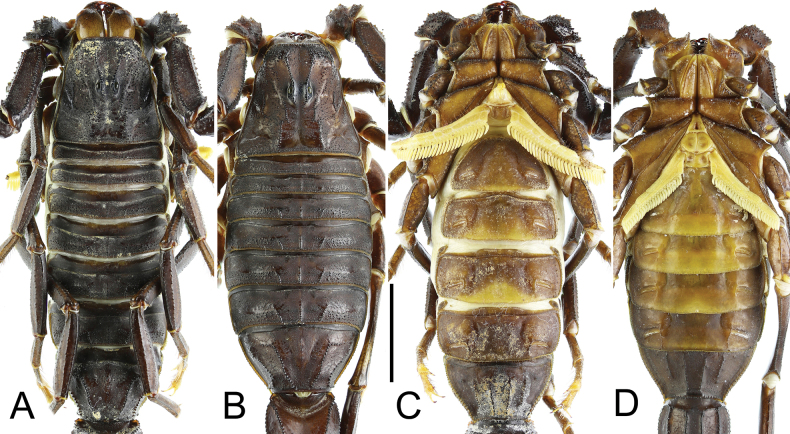
*Androctonus najdensis* sp. nov. male holotype and female paratype. A, C. Male; B, D. Female; A, C. Carapace and mesosoma; B, D. Sternopectinal area and ventral of mesosoma. Scale bar: 10 mm.

(based on male holotype and female paratypes) ***Coloration*** (Figs 1, 2, 4, 6, 8, 10). Base coloration dark brown to blackish brown. Prosoma: carapace dark brown to dark reddish brown in males, and dark reddish brown in females; carinae and granules black. The area surrounding and between median eyes marked with black pigmentation. Chelicerae: manus shiny yellowish brown to dark brown with dark brown reticulations; fingers reddish brown to dark brown, with reddish brown teeth. Pedipalps: femur and patella dark brown dorsally, with black carinae. Chela manus lustrous reddish brown; fingers dark brown posteriorly and dark yellow anteriorly. Denticles black. Legs: tarsi dark yellow; other segments brown. Mesosoma: blackish brown in males; dark brown to dark reddish brown in females. Sternites III–V yellowish brown; poststernites dark yellow medially. Sternite VI pale brown; sternite VII dark brown. Coxae yellowish brown to reddish brown. Pectines pale yellow. Metasoma: ventral surfaces of segments I–V dark brown to black in males and dark brown in females. Lateral and dorsal surfaces reddish brown to brown. Carinae exhibit dark brown to black pigmentation. Vesicle: black ventrally, reddish brown dorsally. Aculeus: reddish brown at base, black at tip.

##### Morphology.

***Prosoma*** (Figs 4A, C, 5A, C): carapace trapezoidal, longer than wide. Carinae strong with coarse granules in males, and moderate granules in females. Intercarinal area densely covered with coarse to medium granules in males, and with medium to minute granules in females. Granules larger and flattened anteriorly, and smaller between posteriomedian, centromedian, and anteromedian carinae. Anterior margin nearly straight and pitted, bearing several stout macrosetae and a row of large, rounded granules. All furrows moderate in depth. Median ocular tubercle located slightly anterior to the centre of carapace. Median eyes separated by a distance equivalent to two ocular diameters, situated in an area bearing several medium-sized granules. There are five pairs of lateral eyes: the first three are moderate in size, aligned, and located above an area with moderate granulation; the last two are vestigial.

Sternum typical for the genus (Type 1), triangular and narrow, longer than wide. The genital operculum longitudinally divided into two semi-oval plates.

***Pectines*** (Figs 4B, D, 5B, D): long (extending well beyond leg IV coxa/trochanter joint in males, slightly surpassing it in females), narrow, and densely setose. Tooth count ranges from 31–33 in males and 23–28 in females. Basal plate heavily sclerotized and wider than long; anterior margin bears a strong median indentation, while posterior margin broadly convex.

***Chelicerae*** (Figs 4A, 4C, 5A, 5C): cheliceral dentition typical for the genus, as defined by Vachon (1963). The surface smooth and shiny, bearing several granules arranged in longitudinal ridges. A distinct cluster of granules is present beneath the movable finger.

***Pedipalps*** (Figs 6–9): trichobothrial pattern Type A, orthobothriotaxic. Dorsal trichobothria on femur arranged in a beta configuration, with *d_2_* located on dorsal surface. Femur pentacarinate, moderately slender, and straight. Dorsointernal, dorsoexternal, and ventrointernal carinae strong, bearing coarse, rounded granules. Ventroexternal carina weak, with moderately sized, spaced, subspinoid granules. Internal median carina weak, with spaced, distinct conical granules. Dorsal intercarinal surface densely covered with granules of various sizes, while ventral intercarinal surface bears fine granules sparsely, with scattered minute granules medially in anterior portion of segment. Patella has eight carinae and moderately stocky and straight. Dorsointernal carina strong and crenulate, bearing three spaced conical granules and terminating distally in a large spinoid granule. Ventrointernal carina strong and granular, terminating in a moderately sized spinoid granule. Dorsal, dorsomedian, and ventromedian carinae strong, bearing coarse, rounded granules. Dorsoexternal and ventroexternal carinae moderate, weakly granular to nearly smooth. Exteriomedian carina weak and smooth. Dorsal intercarinal surface densely covered with fine granules; ventral surface less densely granular. Chela moderately elongated, but manus considerably wider than patella (chela width/patella width = 1.27). Fingers moderately elongated (movable finger/manus length ratio = 1.51) and evenly curved. Manus smooth and lustrous; its internal surface densely granular in males and sparsely granular in females. movable fingers of pedipalps bear 16 rows of denticles, with external and internal accessory denticles and three distal granules. Fixed fingers bear 15 rows of denticles, also with external and internal accessory granules and three distal granules. Trichobothrium *et* located between *dt* and *est*, and proximal to *dt*.

***Legs*** (Figs 14, 15) long, slender, and bear several macrosetae. Basitarsus of legs I–III bears bristle combs, while that of leg IV lacks a bristle comb. Proventral and retroventral basitarsal (pedal) spurs present on legs I and IV, whereas tibial spurs present on legs III and IV. Tarsus of legs I–IV bears spine-like setae ventrally, arranged in two rows.

**Figure 3.**
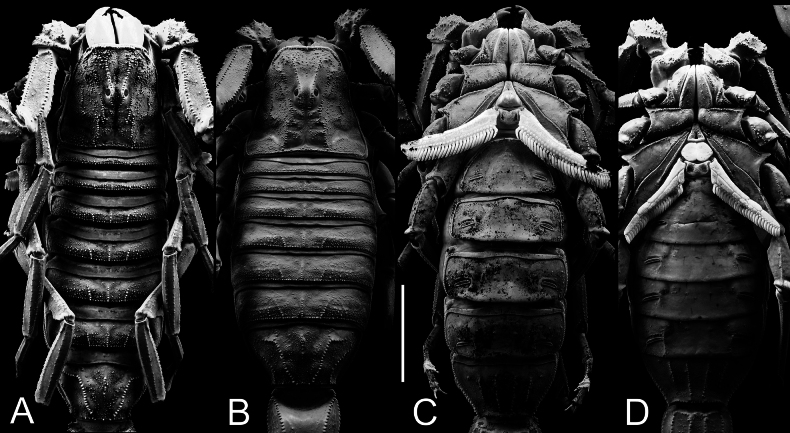
*Androctonus najdensis* sp. nov. male holotype and female paratype, under UV light. A, C. Male; B, D. Female; A, B. Carapace and mesosoma; C, D. Sternopectinal area and ventral of mesosoma. Scale bar: 10 mm.

***Mesosoma*** (Figs 1–3): tergites I–VI possess three moderate, granular carinae (one median and two submedians). Submedian carinae reduced on tergite I. Tergites I–VI densely granular overall. Pretergites finely granular; posttergites coarsely granular, with posterior margins bearing a row of distinct, moderate granules. Tergite VII pentacarinate (with median, submedian, and lateral carinae); median and submedian carinae bear large, rounded granules, while lateral carinae crenulate with spinoid granules. Sternites III–VI smooth with shagreened patches, sparsely setose, and feature elongated, slit-like spiracles. Sternite VI bears a pair of moderate granular carinae; sternite VII has two pairs of moderate granular carinae. Sternite VII finely granular and lacks setae.

**Figure 4.**
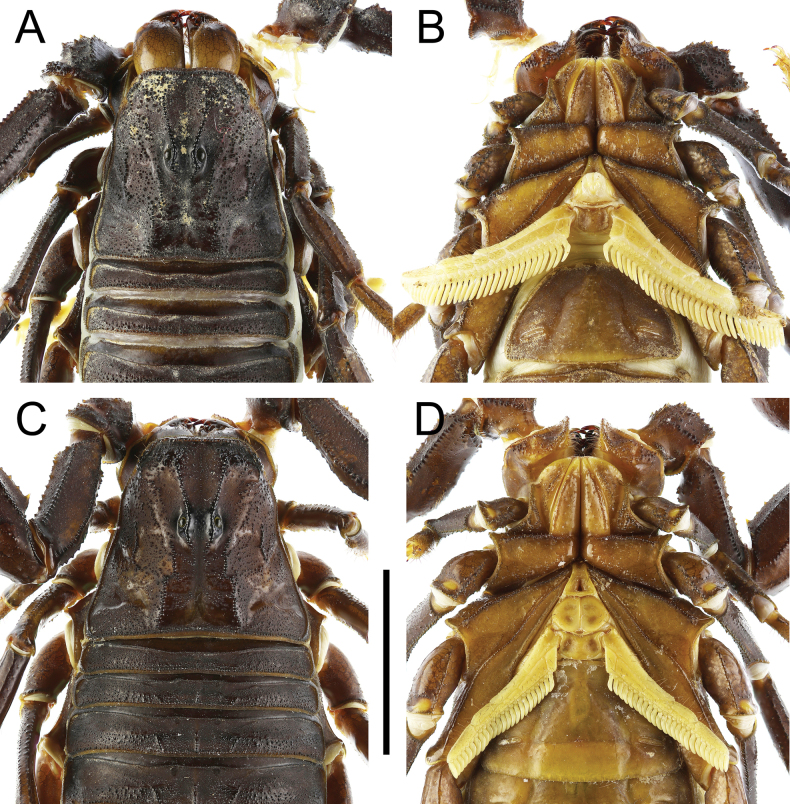
*Androctonus najdensis* sp. nov. male holotype and female paratype. A, B. Male; C, D. Female; A, C. Carapace; B, D. Sternopectinal area. Scale bar: 10 mm.

**Figure 5.**
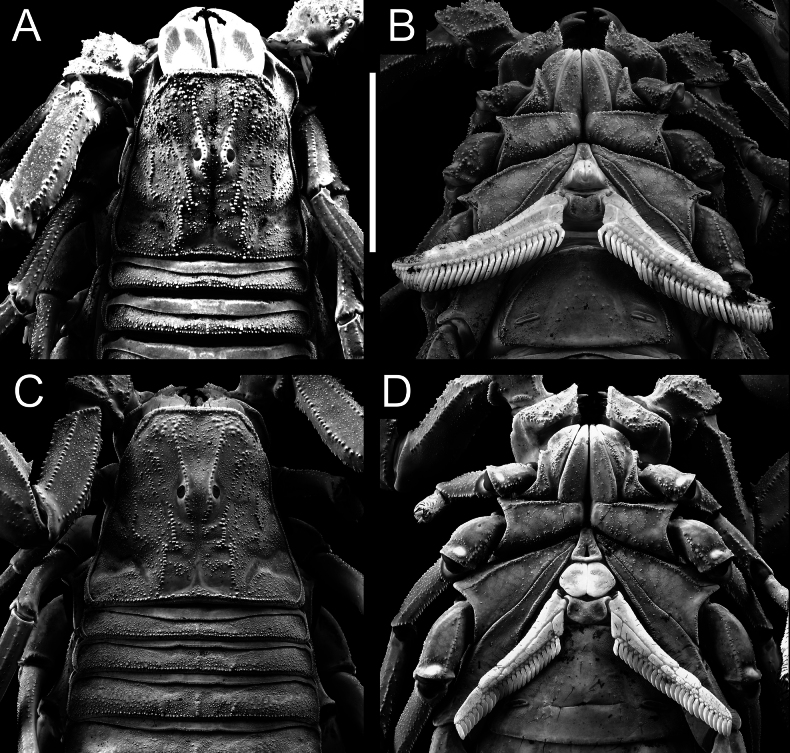
*Androctonus najdensis* sp. nov. male holotype and female paratype, under UV light. A, B. Male; C, D. Female; A, C. Carapace; B, D. Sternopectinal area. Scale bar: 10 mm.

**Figure 6.**
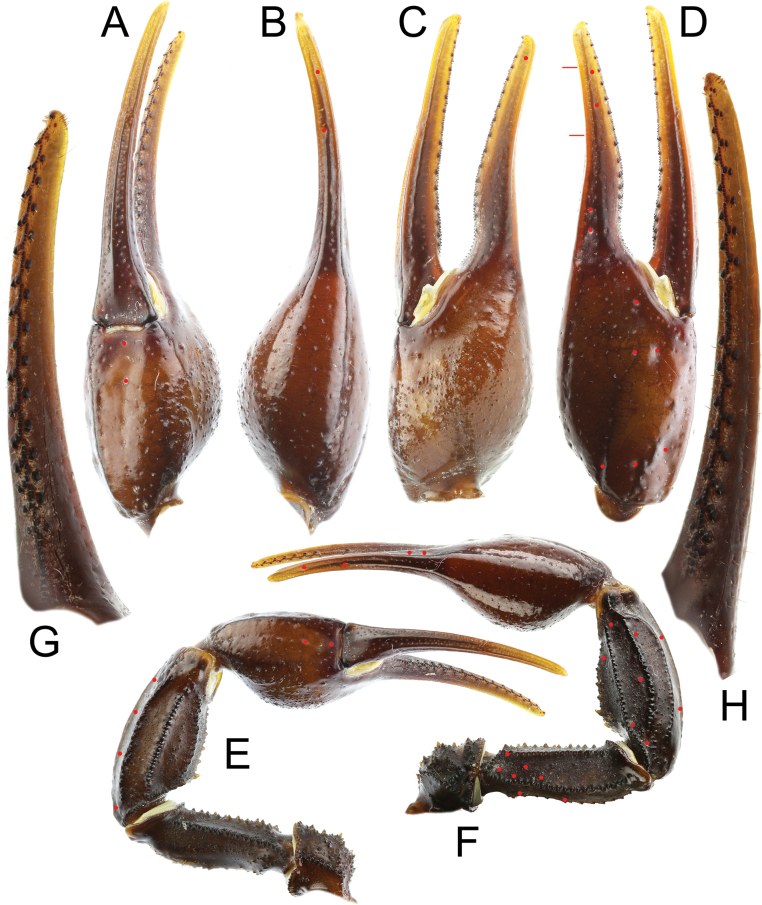
*Androctonus najdensis* sp. nov., pedipalp of male holotype. A. Ventral view of chela; B. Dorsal view of chela; C. Internal view of chela; D. External view of chela; E. Ventral view of pedipalp; F. Dorsal view of pedipalp; G. Fixed finger dentition; H. Movable finger dentition (trichobothrial pattern is indicated by red circles).

**Figure 7.**
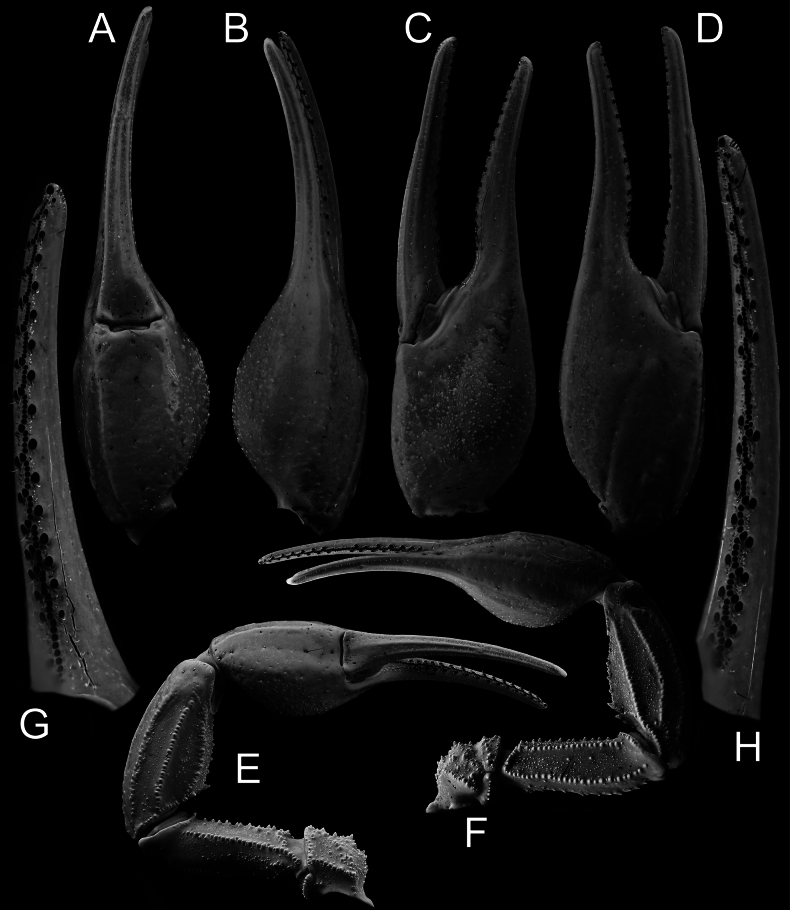
*Androctonus najdensis* sp. nov., pedipalp of male holotype, under UV light. A. Ventral view of chela; B. Dorsal view of chela; C. Internal view of chela; D. External view of chela; E. Ventral view of pedipalp; F. Dorsal view of pedipalp; G. Fixed finger dentition; H. Movable finger dentition.

**Figure 8.**
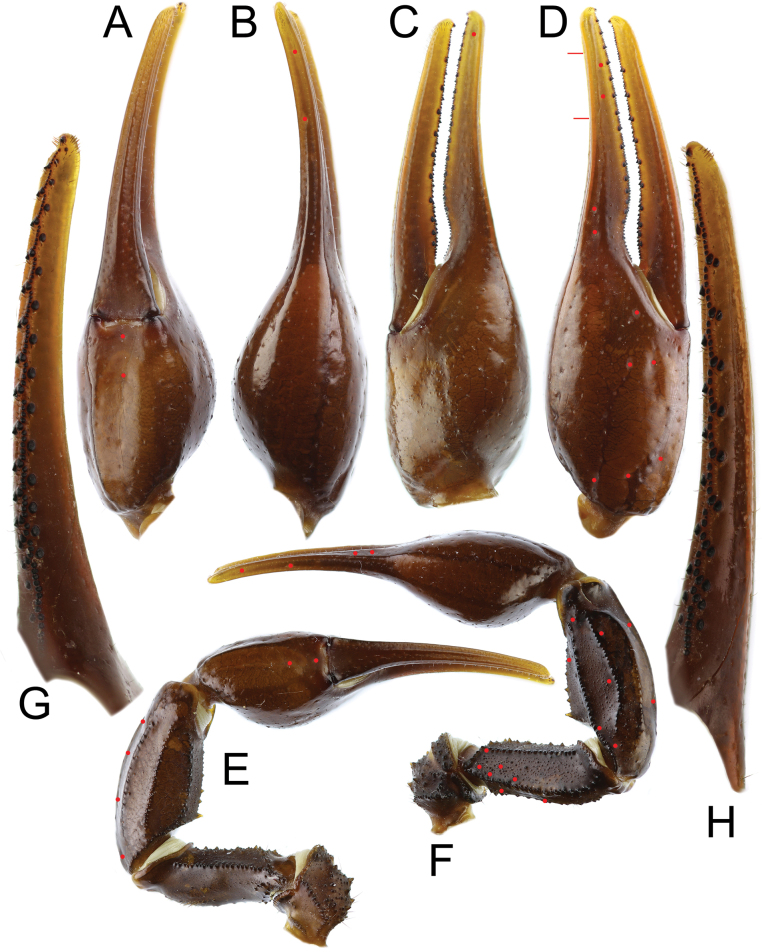
*Androctonus najdensis* sp. nov., pedipalp of female paratype. A. Ventral view of chela; B. Dorsal view of chela; C. Internal view of chela; D. External view of chela; E. Ventral view of pedipalp; F. Dorsal view of pedipalp; G. Fixed finger dentition; H. Movable finger dentition (trichobothrial pattern is indicated by red dots).

**Figure 9.**
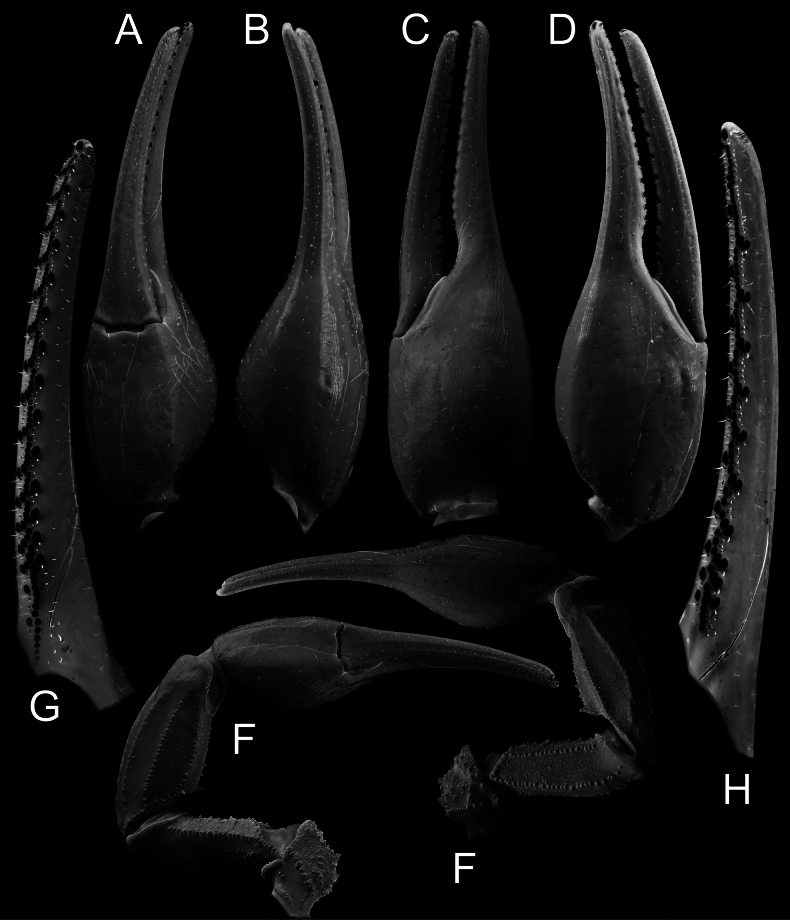
*Androctonus najdensis* sp. nov., pedipalp of female paratype, under UV light. A. Ventral view of chela; B. Dorsal view of chela; C. Internal view of chela; D. External view of chela; E. Ventral view of pedipalp; F. Dorsal view of pedipalp; G. Fixed finger dentition; H. Movable finger dentition.

**Figure 10.**
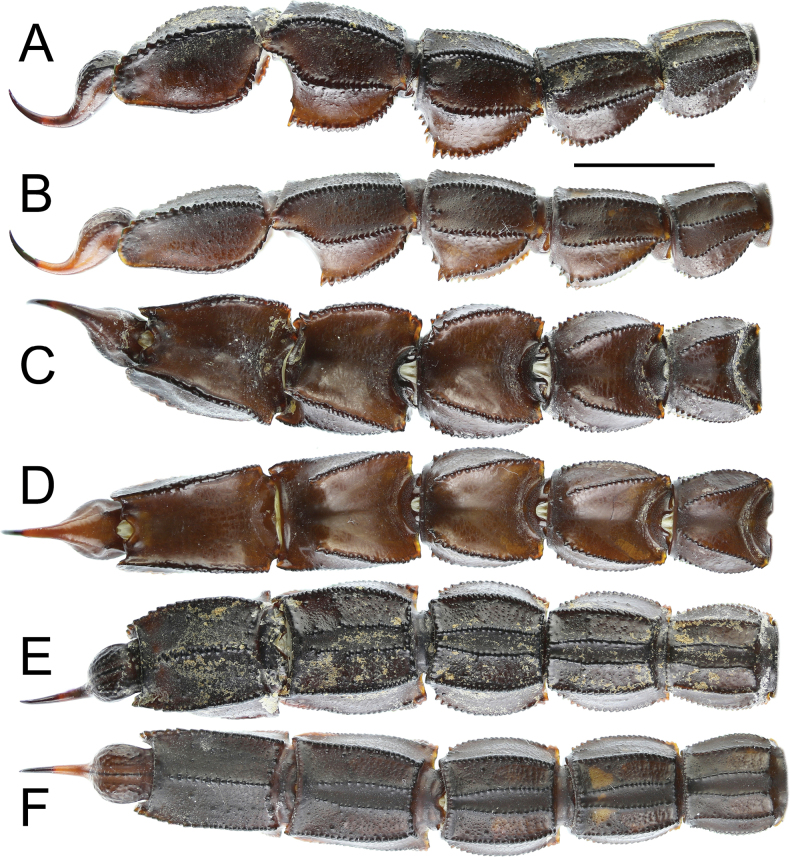
*Androctonus najdensis* sp. nov. metasoma and telson of male holotype and female paratype. A, C, E. Male; B, D, F. Female; A, B. Lateral view; C, D. Dorsal view; E, F. Ventral view. Scale bar: 10 mm.

**Figure 11.**
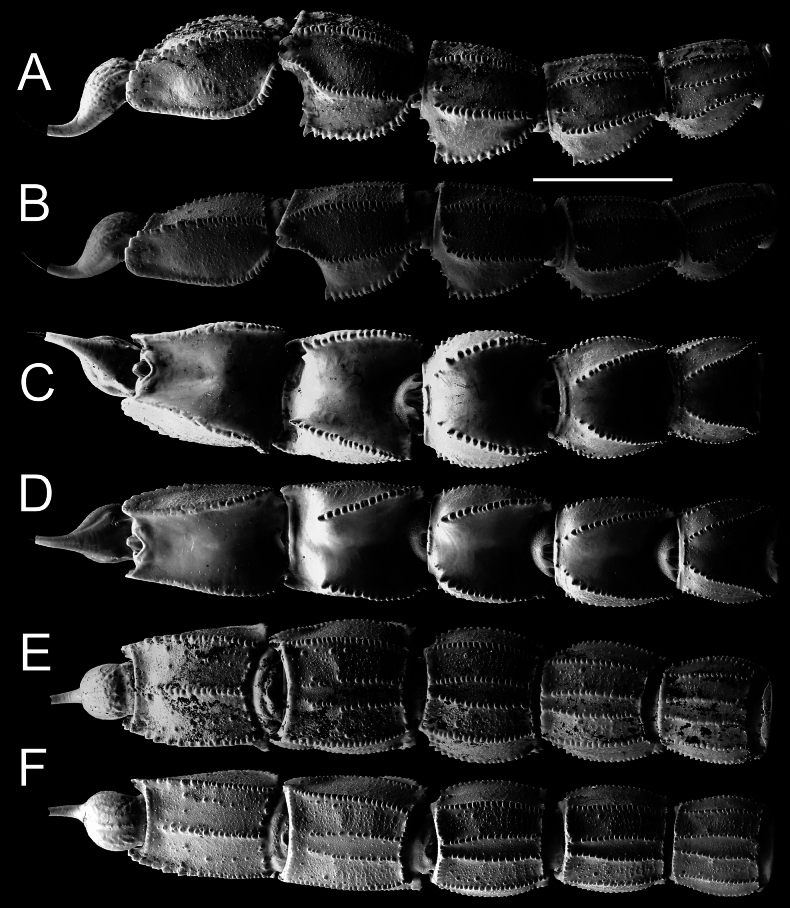
*Androctonus najdensis* sp. nov. metasoma and telson of male holotype and female paratype, under UV light. A, C, E. Male; B, D, F. Female; A, B. Lateral view; C, D. Dorsal view; E, F. Ventral view. Scale bar: 10 mm.

**Figure 12.**
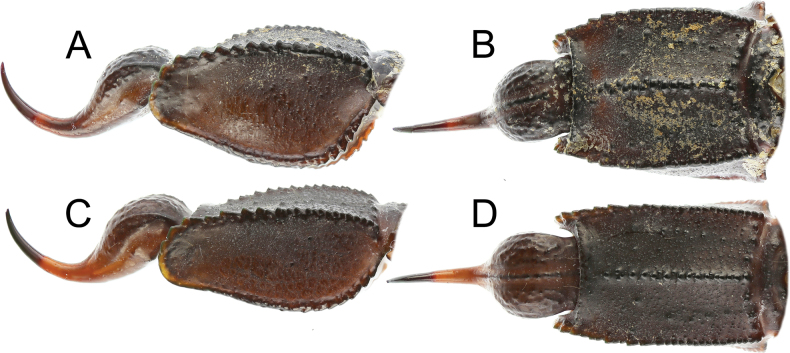
*Androctonus najdensis* sp. nov., metasoma V and telson of male holotype and female paratype. A, B. Male; C, D. Female; A, C. Lateral view; C, D. Ventral view.

**Figure 13.**
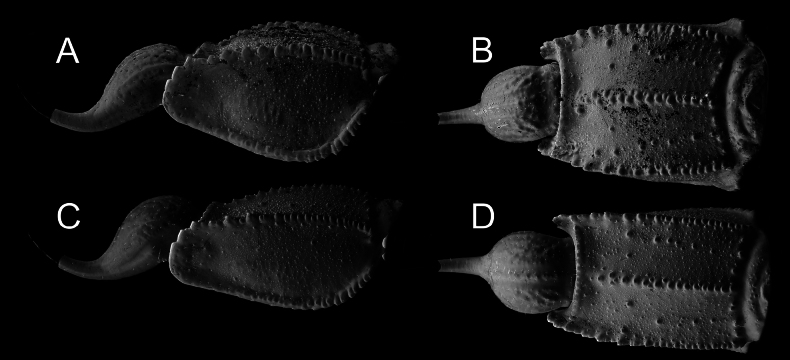
*Androctonus najdensis* sp. nov., metasoma V and telson of male holotype and female paratype, under UV light. A, B. Male; C, D. Female; A, C. Lateral view; B, D. Ventral view.

***Metasoma*** (Figs 10–13): all segments robust, longer than wide, and wider than deep. Segment V considerably stockier in males. Segments I–III bear ten carinae, segment IV has eight, and segment V has five carinae. Lateral inframedian carinae complete, moderate, and granular on segment I; they are incomplete and indistinct on segments II–III, consisting of three granules in the posterior quarter. Dorsolateral carinae strong, with granules gradually increasing in size posteriorly on segments I–IV, serrate on segment I, serrate to dentate or subdentate on segment II, and dentate on segments III–IV. On segment V, dorsolateral carinae strong, serrate posteriorly and smooth anteriorly. Lateral supramedian carinae strong and serrate on segments I–IV, with moderate, rounded granules on segments I and II, and coarse, rounded granules on segments III and IV. Ventrolateral carinae strong on segments I–IV, bearing moderate and rounded granules on segments I and II, and coarse, rounded granules on segments III and IV. On segment V, they are strong, with somewhat large granules that lack enlarged denticles and increase slightly in size posteriorly. Ventral submedian carinae moderate on segments I–IV, bearing moderately sized rounded granules. Ventromedian carina on segment V moderate, with similar rounded granules. Anal arch bears two lateral rounded lobes, the inferior one being twice as large as the superior and slightly split. Metasoma very sparsely setose. Dorsal intercarinal areas finely granular medially on segments I and II, and smooth without granulation on segments III–V. Lateral and ventral surfaces rough and densely covered with fine granules on segments I–V. Dorsal furrow moderately deep and wide on all segments. Telson slender and elongated. Vesicle small, somewhat elongated, with a wrinkled tegument but smooth surface, and shows distinct to obsolete ventromedian carinae. Aculeus long, approximately equal in length to vesicle, and moderately curved.

**Figure 14.**
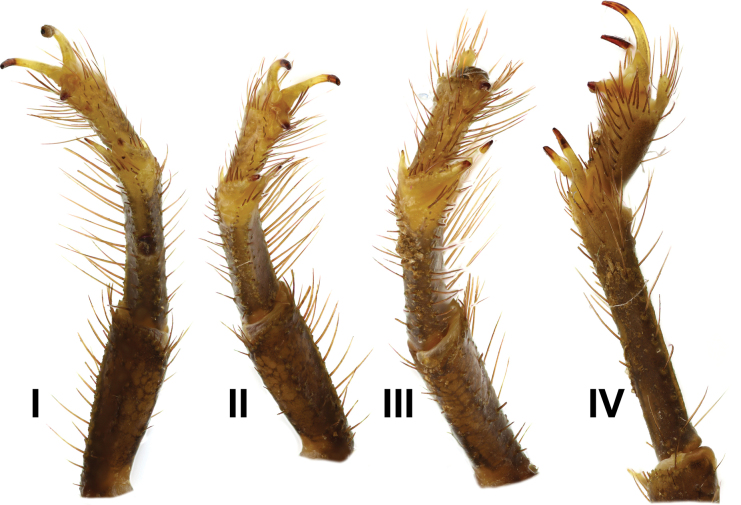
*Androctonus najdensis* sp. nov., male holotype, right legs I–IV.

**Figure 15.**
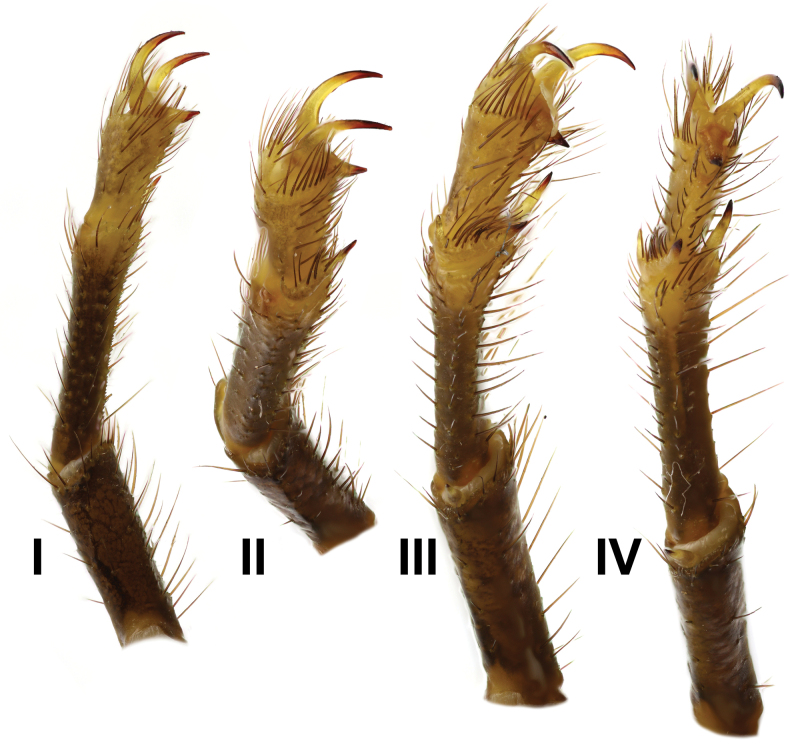
*Androctonus najdensis* sp. nov., female paratype, right legs I–IV.

##### Etymology.

The new species is named after the Najd Plateau, located in the central region of the Arabian Peninsula.

##### Affinities.

*Androctonus najdensis* sp. nov. can be distinguished from other *Androctonus* species occurring in the Middle East, Iran, and Turkey by the following characters.

General coloration of *A. najdensis* sp. nov. is dark brown to blackish brown, whereas in *A. australis*, *A. amoreuxi*, *A. sistanus*, and *A. tihamicus*, it is pale brown to yellow. *Androctonus najdensis* sp. nov. has a wide manus, while *A. bicolor* and *A. minaeus* have a slender manus.

In *A. crassicauda* and *A. orientalis*, the fifth metasomal segment in males is elongated, and most of the dorsolateral carinae are serrate. In contrast, in *A. najdensis* sp. nov., the fifth metasomal segment in males is stocky, and only a small portion of the dorsolateral carina is serrate. Besides, in *A. crassicauda* and *A. orientalis* the dorsolateral carina of the fourth segment is distinctly dentate, whereas it is serrate in *A. najdensis* sp. nov.

Additionally, the ventrolateral carinae of fifth metasomal segment in *A. najdensis* sp. nov. lack enlarged denticles, whereas in *A. ammoneus*, *A. azerianus*, *A. barahoeii*, *A. caspius*, *A. ishtar*, *A. kunti*, *A. sumericus*, *A. transcaucasicus* and *A. turkiyensis* ventrolateral carinae bear enlarged or somewhat large denticles.

Coloration is brown in *A. ammophilus* and *A. omanensis* whereas it is black in *A. najdensis* sp. nov.

The lateral surface of segment V of the metasoma is smooth and the ventrolateral carinae of segment V bear dentate granules posteriorly in *A. omanensis* whereas the lateral surface of segment V is finely granular and the ventrolateral carinae of segment V are subconical, and do not bear dentate granules in *A. najdensis* sp. nov.

The seventh sternite is smooth in *A. ammophilus* whereas in *A. najdensis* sp. nov. it is finely granular.

### ﻿Key to species of the genus *Androctonus* in the Middle East, Turkey, and Iran

**Table:** 

1	Coloration reddish brown, brown, or black	**2**
–	Coloration yellow	**17**
2	Chela stocky, fingers short	**3**
–	Chela elongated, fingers long	**19**
3	Ventrolateral carinae on segment V with enlarged denticles	**4**
–	Ventrolateral carinae on segment V without large denticles	**12**
4	Intercarinal area of carapace covered with small or medium sized granules. Chela has a distinct gap between fingers	***A. sumericus***
–	Intercarinal area of carapace covered with coarse granules. Chela exhibits an indistinct gap between fingers.	**5**
5	Metasoma II with lateral inframedian carinae incomplete but developed on the posterior ¾	***A. azerianus***
–	Metasoma II with lateral inframedian carinae absent, represented by only 2–5 denticles on the posterior part	**6**
6	Pedipalp chela internal surface densely or moderately granular with fine granules. Chela manus wide in males and narrow in females	**7**
–	Pedipalp chela internal surface smooth, with only several fine granules. There is no noticeable difference in chela shapes between the sexes	**8**
7	Pedipalp chela internal surface densely granular. Ventrolateral carinae on segment V with 1 or 2 separated, enlarged denticles	***A. barahoeii***
–	Pedipalp chela internal surface moderately granular. Ventrolateral carinae on segment V with 2 fixed, enlarged denticles	***A. ishtar***
8	Metasoma lateral surfaces rough	**9**
–	Metasoma lateral surfaces smooth	**10**
9	Ventrolateral carinae of segment V bear conspicuous 2 or 3 subconical denticles	***A. turkiyensis***
–	Ventrolateral carinae of segment V bear 2 or 3 less conspicuous subspinoid denticles	***A. ammoneus***
10	Chela finely granulated, tibial spurs on legs III and IV moderate	***A. kunti***
–	Chela smooth, tibial spurs on legs III and IV strong	**11**
11	Pedipalp chela length/width ratio 3.7 (male) to 4.6 (female)	***A. transcaucasicus***
–	Pedipalp chela length/width ratio 4.4–4.7 in both sexes, without sexual dimorphism	***A. caspius***
12	Coloration reddish brown or yellowish brown	***A. tihamicus***
–	Coloration brown or black	**13**
13	Metasomal segment V of metasoma stocky especially in males. Only a small portion of dorsolateral carina of segment V serrate. Dorsolateral carina of segment IV serrate	**14**
–	Metasomal segment V of metasoma especially in males elongate. Most of dorsolateral carina of segment V serrate. Dorsolateral carina of segment IV distinctly dentate	**16**
14	Lateral surface of metasomal segment V smooth. Ventrolateral carinae of segment V bear dentate granules at posterior portion	***A. omanensis***
–	Lateral surface of metasomal segment V finely granular. Ventrolateral carinae of segment V bear subconical, do not bear dentate granules	**15**
15	Coloration is black. Sternite VII finely granular	***A. najdensis* sp. nov.**
–	Coloration is brown. Sternite VII smooth	***A. ammophilus***
16	Sternites finely granular	***A. crassicauda***
–	Sternites smooth	***A. orientalis***
17	Metasomal segments IV and -V and telson yellow	**18**
–	Metasomal segments IV and V and telson black	***A. sistanus***
18	Metasomal segments stocky	***A* . *australis***
–	Metasomal segments elongated	***A. amoreuxi***
19	Chela length/width ratio 7.27 in male and 8.24 in female	***A. minaeus***
–	Chela length/width ratio 5.65 in male and 6.45 in female	***A. bicolor***

### ﻿Ecology

Most specimens were collected from the Najd Plateau region of central Saudi Arabia and from wadis surrounding the Tuwayq Escarpment, with additional samples obtained from the Eastern Province and Ha’il. Najd is a plateau that ranges in elevation from 762 to 1,525 meters and slopes eastward. Notable topographic features include the Aja and Salma mountains, Jabal Shammar, and the Tuwayq mountain range. Dry wadis, such as Wadi Hanifa and Wadi Na’am, play an important role in retaining rainwater. The recorded elevation range for the species is 20–800 meters, with the lowest occurrences near the eastern coast and the highest on the Najd Plateau. The new species is likely lapidicolous, inhabiting areas under rocks and debris in wadis within both sedimentary and igneous hills and mountains, as well as animal burrows in sandy desert soils.

### ﻿Genetic analysis

**Figure 16.**
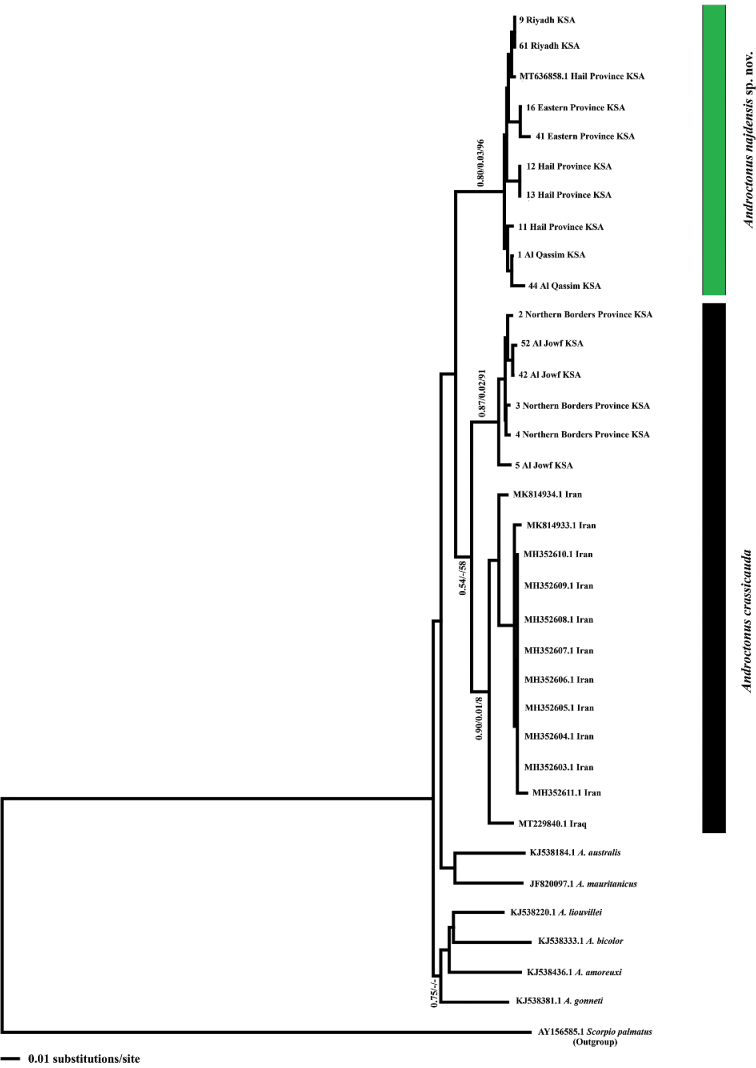
Neighbour-joining (NJ) phylogenetic tree of *Androctonus* species based on COI sequences from Saudi Arabia and the Middle East. Numbers above and below branches indicate Bayesian posterior probabilities/ NJ distance values/ maximum parsimony bootstrap values.

The phylogenetic tree topologies recovered by the analysis of the COI data set resulted in an identical topology between the Maximum-parsimony and the neighbour-joining trees (Fig. 16). However, the general topology of the maximum-parsimony tree was slightly different from those obtained by Bayesian inference analyses. *Androctonus najdensis* sp. nov. forms a monophyletic clade distinct from *A. crassicauda* s.l. (from Saudi Arabia, Iraq, and Iran) and other related *Androctonus* species analysed (Fig. 16). Also, the new species differs from *A. crassicauda* by a raw genetic distance of 7.0–8.0%, and from other *Androctonus* species by 10–11.0% (Table 3).

**Table 3.** The uncorrected *p*-distance of the sequence divergence of COI mtDNA sequences between *Androctonus* samples included in this study.

	1	2	3	4	5	6	7	8	9	10	11	12
1. *A. najdensis* sp. nov. KSA		0.01	0.01	0.01	0.01	0.01	0.01	0.01	0.01	0.01	0.01	0.02
2. *A. crassicauda* _Northern_Border_KSA	0.07		0.00	0.01	0.01	0.01	0.01	0.01	0.01	0.01	0.01	0.02
3. *A. crassicauda* _Al_Jowf_KSA	0.07	0.01		0.01	0.01	0.01	0.01	0.01	0.01	0.01	0.01	0.02
4. *A. crassicauda* _Iran	0.08	0.05	0.05		0.01	0.01	0.01	0.01	0.01	0.01	0.01	0.02
5. *A. crassicauda* _Iraq	0.08	0.05	0.05	0.03		0.01	0.01	0.01	0.01	0.01	0.01	0.02
6. *A. liouvillei*	0.11	0.10	0.10	0.09	0.09		0.01	0.01	0.01	0.01	0.01	0.02
7. *A. gonneti*	0.11	0.10	0.10	0.09	0.09	0.08		0.01	0.01	0.01	0.01	0.02
8. *A. australis*	0.10	0.10	0.10	0.10	0.09	0.11	0.10		0.01	0.01	0.01	0.02
9. *A. mauritanicus*	0.10	0.10	0.10	0.10	0.10	0.09	0.11	0.08		0.01	0.01	0.02
10. *A. amoreuxi*	0.11	0.10	0.10	0.11	0.10	0.07	0.09	0.10	0.10		0.01	0.02
11. *A. bicolor*	0.11	0.11	0.11	0.11	0.11	0.08	0.10	0.10	0.09	0.10		0.02
12. Outgroup	0.63	0.64	0.64	0.65	0.64	0.64	0.64	0.64	0.66	0.64	0.65	

**Figure 17.**
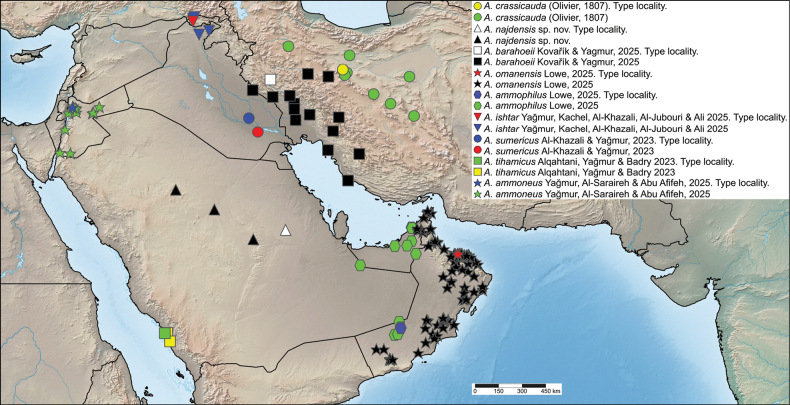
A map showing the distribution of *A. ammoneus*, *A. ammophilus*, *A. barahoeii*, *A. crassicauda*, *A. ishtar*, *A. najdensis* sp. nov., *A. omanensis*, *A. sumericus* and *A. tihamicus* in Iraq, Iran, Jordan, Oman, Saudi Arabia, and United Arab Emirates.

## ﻿Discussion

Several authors have employed mitochondrial genes to identify cryptic species within the genus *Androctonus*, including Ben Ali et al. (2000), Othmen et al. (2009), Ozkan et al. (2010), Desouky and Alshammari (2010), Coelho et al. (2014), and Alqahtani et al. (2022a, b, 2023). In the present study, we conducted a molecular phylogenetic analysis using the COI mitochondrial gene for this purpose. The results revealed a genetic divergence (p-distance = 0.07–0.08) between *A. najdensis* sp. nov. and populations of *A. crassicauda* from northern Saudi Arabia, Iraq, and Iran. Additionally, the new species differed from North African *Androctonus* species by a p-distance of 0.10–0.11 (Table 3). The combined molecular and morphological evidence strongly supports the recognition of the Najd Plateau population as a distinct species.

Several previous studies (Vachon 1979; Hendrixson 2006; Al-Asmari et al. 2009, 2013; Alqahtani et al. 2019) have reported *A. crassicauda* from various localities and provided general illustrations. Vachon (1979), Al-Asmari et al. (2009), and Alqahtani et al. (2019) illustrated specimens from Riyadh, Ha’il, and southwestern Saudi Arabia, respectively. However, Hendrixson (2006) did not specify the locality of the illustrated specimen. These specimens show morphological similarities to *A. najdensis* sp. nov., particularly in their dark coloration and the absence of enlarged denticles on the fifth metasomal segment. Notably, specimens of the newly described species were collected from Riyadh and Ha’il, suggesting that the individuals illustrated in previous studies may in fact belong to *A. najdensis* sp. nov.

## Supplementary Material

XML Treatment for Androctonus najdensis

## ﻿Additional information

### Use of AI

No use of AI was reported.

### Author ORCIDs

Ersen Aydın Yağmur https://orcid.org/0000-0002-0396-3975

Abdulaziz R. Alqahtani https://orcid.org/0000-0003-2682-7965

Ahmed Badry https://orcid.org/0000-0002-2610-2019

### Data availability

All of the data that support the findings of this study are available in the main text.
